# Oxalate Pushes Efficiency of CsPb_0.7_Sn_0.3_IBr_2_ Based All‐Inorganic Perovskite Solar Cells to over 14%

**DOI:** 10.1002/advs.202106054

**Published:** 2022-02-12

**Authors:** Weihai Zhang, Heng Liu, Xingnan Qi, Yinye Yu, Yecheng Zhou, Yu Xia, Jieshun Cui, Yueqing Shi, Rui Chen, Hsing‐Lin Wang

**Affiliations:** ^1^ Department of Materials Science and Engineering South University of Science and Technology Shenzhen 518055 China; ^2^ School of Materials Science and Engineering Sun Yat‐sen University Guangzhou 510275 China; ^3^ School of Physics and Astronomy University of Birmingham Edgbaston Birmingham B152TT UK; ^4^ Department of Electrical and Electronic Engineering South University of Science and Technology Shenzhen 518055 China

**Keywords:** all‐inorganic perovskites, bromine‐rich, lead‐reduced, mitigative oxidation, oxalate

## Abstract

All‐inorganic CsPbIBr_2_ perovskite solar cells (PSCs) have recently gained growing attention as a promising template to solve the thermal instability of organic–inorganic PSCs. However, the relatively low device efficiency hinders its further development. Herein, highly efficient and stable CsPb_0.7_Sn_0.3_IBr_2_ compositional perovskite‐based inorganic PSCs are fabricated by introducing appropriate amount of multifunctional zinc oxalate (ZnOX). In addition to offset Pb and Sn vacancies through Zn^2+^ ions incorporation, the oxalate group can strongly interact with undercoordinated metal ions to regulate film crystallization, delivering perovskite film with low defect density, high crystallinity, and superior electronic properties. Correspondingly, the resulting device delivers a champion efficiency of 14.1%, which presents the highest reported efficiency for bromine‐rich inorganic PSCs thus far. More importantly, chemically reducing oxalate group can effectively suppress the notorious oxidation of Sn^2+^, leading to significant enhancement on air stability.

## Graphical abstract

Highly efficient CsPb_0.7_Sn_0.3_IBr_2_ perovskite solar cells (PSCs) are achieved via introducing zinc oxalate (ZnOX) as additive. ZnOX not only reduces the metal ions related trap states but also modulates film crystallization, resulting in a record device efficiency of 14.1%. In particular, chemically reducing oxalate can effectively suppress the oxidation of Sn^2+^, contributing to devices with much improved long‐term air stability.

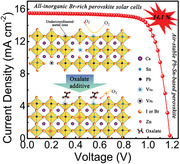

## 1 Introduction

Thermal instability of volatile organic cations (e.g., methylammonium (MA^+^) and formamidinium (FA^+^)) based perovskite solar cells (PSCs) has become a major concern for further commercialization in recent years.^[^ ^1^, ^2^, ^3^ ^]^ One promising solution is to completely replace these organic cations with inorganic cations, such as cesium (Cs^+^) and rubidium (Rb^+^) ions, to develop all inorganic PSCs.^[^ ^4^, ^5^, ^6^ ^]^ Due to the absence of release or decomposition of organic cations, inorganic perovskites can maintain their crystal structure and thus device performance at temperatures above 400 °C, showing great potential toward practical application.^[^ ^7^, ^8^ ^]^

At present, widely studied all inorganic perovskites include CsPbI_3_,^[^ ^9^, ^10^ ^]^ CsPbBr_3_,^[^ ^11^, ^12^ ^]^ and CsPbI_3−_ *_y_* Br*_y_*,^[^ ^13^, ^14^, ^15^ ^]^ where *y* is the molar ratio of bromine in the precursor. Among them, black phase *α*‐CsPbI_3_ perovskite with a bandgap (*E*_g_) of 1.73 eV gains broad interests as an ideal top absorber candidate for perovskite tandems.^[^ ^16^ ^]^ Unfortunately, the notorious thermodynamic instability of *α*‐CsPbI_3_ to photoinactive nonperovskite phase (*δ*‐CsPbI_3_) at room temperature has hindered its further development toward commercial applications. In contrast, by replacing iodine with bromine, CsPbBr_3_ perovskite exhibits superior phase stability in ambient environment due to the increased octahedral factor.^[^ ^17^, ^18^ ^]^ However, its wide *E*_g_ of 2.3 eV has led to substantial optical losses, limiting the short circuit current of the corresponding photovoltaic devices. Alternatively, mixed‐halide CsPbI_3−_ *_y_* Br*_y_* perovskites are feasible analogs to balance the trade‐off between phase stability and bandgap. When *y* comes to 1, CsPbI_2_Br perovskite with a *E*_g_ of 1.9 eV was achieved. Despite the exciting device efficiency of over 17%,^[^ ^19^ ^]^ CsPbI_2_Br is still structurally unstable and can easily transform into *δ*‐phase under prolonged exposure to high humidity. By further increasing Br ratio, CsPbIBr_2_ perovskite has been considered as a potential candidate for inorganic PSCs due to its well‐balanced *E*_g_ of 2.05 eV and significantly improved stability against temperature, humidity, and light irradiation. However, the power conversion efficiency (PCE) of pure CsPbIBr_2_ perovskite‐based PSCs lags far behind its iodine‐rich counterparts.^[^ ^20^ ^]^ Therefore, novel inorganic halide perovskites with better trade‐off between bandgap and stability need to be rationally designed.

Apart from X site halide manipulation, B site metal ion substitution in an ABX_3_ inorganic perovskite has been reported as an effective strategy to adjust optoelectronic properties of the material. For instance, there are a few studies that use metal ions, such as Sn^2+^, Mn^2+^, Zn^2+^, Sr^2+^, Cu^2+^, and Ge^2+^, to partially substitute Pb^2+^ in perovskites, demonstrating stabilized photoactive phase and enhanced device performance.^[^ ^21^, ^22^, ^23^, ^24^, ^25^, ^26^, ^27^ ^]^ In particular, substituting Pb^2+^ with Sn^2+^ in the lead halide perovskites can significantly reduce the bandgap, thus broadening the effective light absorption region for high performance PSCs. Liang et al. fabricated the first Pb–Sn based inorganic perovskite film with a chemical composition of CsPb_0.9_Sn_0.1_IBr_2_ via a conventional two‐step sequential method in ambient air. The resulting device yields a PCE of 11.33% with a high open‐circuit voltage (*V*_oc_) of 1.26 V.^[^ ^28^ ^]^ Afterward, Li et al. deposited CsPb_1−_ *_x_* Sn*_x_* IBr_2_ perovskite films by employing one‐step antisolvent method, and the resulted films exhibited tunable *E*_g_ from 2.04 to 1.78 eV with *x* value ranged from 0 to 0.25. The corresponding champion device showed a remarkable PCE of 11.53% with much improved phase and light stability.^[^ ^22^ ^]^ These studies reveal the potential of mixed Pb–Sn based inorganic perovskite as favorable absorber for efficient and stable all‐inorganic PSCs. However, two main problems remain to be resolved. First, uncontrollable crystallization rate due to the high Lewis acidity of Sn^2+^ hinders the formation of uniformed perovskite films, leading to poor device performance. Second, fast oxidation of Sn^2+^ to Sn^4+^ leads to high density of trap states, resulting in significant carrier recombination in the perovskite film.^[^ ^29^ ^]^

In this work, we successfully fabricated CsPb_1−_ *_x_* Sn*_x_* IBr_2_ based PSCs with greatly enhanced air stability and photovoltaic performance by employing zinc oxalate (ZnC_2_O_4_, abbreviated as ZnOX) as additive during film formation. On one hand, bivalent Zn^2+^ ions can offset part of Pb and Sn vacancies (V_Pb_ and V_Sn_), resulting in film with low defect density of states. On the other hand, the carboxyl group of oxalates can strongly coordinate with metal ions at the surface to slow down the formation of perovskite nuclei, thus regulating film crystallization. With this multifunctional additive, the resulting perovskite films show excellent surface morphology with micrometer‐sized crystalline grains, leading to long carrier life time, low defect density, and high electron mobility. Consequently, with 2% optimal ZnOX addition, the as fabricated devices based on CsPb_0.7_Sn_0.3_IBr_2_ perovskite yield a record PCE of 14.1%. More importantly, chemically reducing oxalate group can effectively suppress the oxidation of Sn^2+^ ions, contributing to devices with much improved long‐term stability.

## 2 Results and Discussion

**Figure 1:**
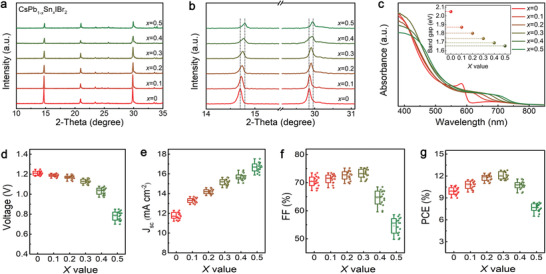
a) XRD patterns of CsPb_1−_ *_x_* Sn*_x_* IBr_2_ perovskite film with different *x* value. b) Closer inspection of the (100) and (200) diffraction peaks. c) UV–vis absorption spectra and bandgap (inset) of perovskite films. d–g) Statistical *V*_oc_, *J*_sc_, FF, and PCE of 15 independent PSCs based on different perovskite films.

Mixed Pb–Sn based inorganic CsPb_1−_ *_x_* Sn*_x_* IBr_2_ perovskite films were fabricated using a seed‐assisted growth (SAG) method as reported in our previous work.^[^ ^15^ ^]^ To obtain the target films with different *x* value, appropriate amount of CsSnIBr_2_ stock solution was added to the CsPbIBr_2_ solution to prepare the precursor, and their ratio was carefully controlled. The crystalline structure of the resulting perovskites was studied by conducting X‐ray diffraction (XRD). As shown in **Figure** **1**a, all films show several main diffraction peaks at 14.7°, 20.9°, and 29.8°, which correspond to (100), (110), and (200) planes of CsPb_1−_ *_x_* Sn*_x_* IBr_2_, respectively. Besides, it is noted that the diffraction intensity of the peaks gradually decreased with the increase of the *x* value. This indicates an inferior crystallization after Sn incorporation due to the fact that high Lewis acidity of Sn^2+^ hinders the formation of highly crystallized perovskite films as mentioned above.^[^ ^29^ ^]^ Additionally, a close inspection of the (100) and (200) perovskite planes shown in Figure 1b reveals a peak shift toward higher angles along with the increase of *x* value, which is attributed to the shrinkage of the perovskite lattice due to the incorporation of smaller radius Sn atom. Figure 1c shows the UV–vis absorption spectra of the perovskites, in which a gradual redshift of the absorption onset from 605 to 750 nm was observed with the increase of the *x* value from 0 to 0.5. Based on the plots of (*αhυ*)^2^ versus photon energy (*hυ*) converted from the UV–vis absorption spectra (Figure S1, Supporting Information), it is calculated that the bandgap of the perovskites decreased from 2.05 eV (*x* = 0) to 1.65 eV (*x* = 0.5), as shown in the inset of Figure 1c. The lower *E*_g_ of CsPb_1‐_ *_x_* Sn*_x_* IBr_2_ than pure CsPbIBr_2_ makes it possible to absorb more photon for higher photocurrent, leading to improved device performance.

Figure 1d–g gives the statistical distribution of the open circuit voltage (*V*_oc_), short current density (*J*_sc_), fill factor (FF), and PCE of 15 independent PSCs based on CsPb_1−_ *_x_* Sn*_x_* IBr_2_ perovskites as a function of *x* value. Corresponding photocurrent density–voltage (*J*–*V*) curves and photovoltaic parameters of the champion devices are presented in Figure S2 (Supporting Information) and Table S1 (Supporting Information), respectively. Clearly, the devices show a decrease in *V*_oc_ and increase in *J*_sc_ with increasing *x* value, which can be ascribed to the reduction of *E*_g_. In addition, the FF reveals an incremental increase at first reaching a maximum at *x* = 0.3, but sharply decreased with a further increase of the *x* value. Consequently, the PCE of the devices initially increased and then decreased. The highest PCE of 12.8% derived from CsPb_0.7_Sn_0.3_IBr_2_ based device suggests that the optimal mole ratio of Sn located at *x* = 0.3.

**Figure 2:**
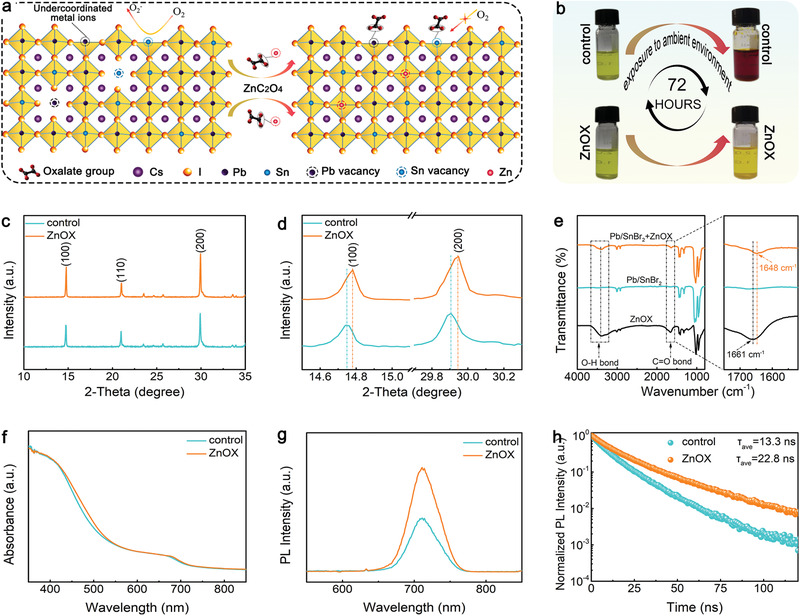
a) Schematic illustrating the functions of ZnOX. b) Photographs of showing the mitigation of oxidation of Sn^2+^ to Sn^4+^ in ambient air after ZnOX introduction. c) XRD patterns of control and ZnOX films. d) Enlarged (100) and (200) XRD peaks. e) FTIR spectra and fingerprint regions of pure ZnOX, Pb/SnBr_2_, and Pb/SnBr_2_+ZnOX in DMSO. f) UV–vis absorption spectra, g) steady‐state photoluminescence (PL) spectra, and h) time‐resolved photoluminescence (TRPL) spectra of control and ZnOX film.

To further advance the performance of Pb–Sn based inorganic perovskite devices, ZnOX was introduced to modulate film crystallization and suppress Sn^2+^ oxidation. As illustrated in **Figure** **2**a, during the formation of CsPb_0.7_Sn_0.3_IBr_2_ perovskite film (denoted as control hereafter), abundant Pb and Sn vacancies (V_Pb_ and V_Sn_) and undercoordinated metal ions are formed due to the different crystallization rate of Pb and Sn‐based perovskite.^[^ ^30^ ^]^ These defects can induce the formation of deep level traps which are major nonradiative recombination centers, deteriorating device performance.^[^ ^31^, ^32^ ^]^ After ZnOX introduction (referred to as ZnOX from hereon), part of V_Pb_ and V_Sn_ can be offset by Zn^2+^ ions incorporation. Besides, as a bidentate capping ligand, chemically reducing oxalate can strongly coordinate on the surface of perovskites. This interaction not only slows down film crystallization but also mitigates the oxidation of Sn^2+^.^[^ ^33^ ^]^ Figure 2b shows the photographs of the control and ZnOX perovskite precursor solutions at different stages. Both as‐prepared perovskite solutions present a semitransparent light‐yellow color. Interestingly, after 72 h exposure to air, the control precursor solution color turns reddish‐brown, while the ZnOX solution reveals chrome yellow. This distinct color change indicates a different oxidation process from Sn^2+^ to Sn^4+^ in the solutions, which can be explained through the following equations. With exposure to air, the Sn^2+^ ions in the control precursor can be easily oxidized to Sn^4+^ ions through Equation (1), leading to a color change from light‐yellow to reddish‐brown.^[^ ^34^ ^]^ In contrast, in the ZnOX solution, the oxidation of Sn^2+^ ions was effectively suppressed due to the existence of chemically reducing oxalate group through Equation (2). Moreover, the reaction product is CO_2_ that escapes from the solution without any contamination, resulting in pure Pb–Sn based inorganic perovskite.

(1) $$2Sn^{2+}+O_{2}\to 2Sn^{4+}+2O^{2-}$$

(2) $${\left(C_{2}O_{4}\right)}^{2-}+Sn^{4+}\to 2CO_{2}↑+Sn^{2+}$$

To investigate the effect of ZnOX addition, comparative studies on crystalline structure and optical properties of the resulting films were conducted. As shown in Figure 2c, the identical diffraction peaks, which correspond to perovskite planes, indicate that ZnOX did not alter the crystalline structure of perovskite. However, the stronger diffraction peak intensity suggests that ZnOX perovskite film shows an enhanced crystallinity. In addition, the enlarged (100) and (200) perovskite planes shown in Figure 2d reveals a peak shift toward higher angles for ZnOX film, implying that Zn^2+^ with smaller ion radius was incorporated into the perovskite lattice sites.^[^ ^35^, ^36^ ^]^ Figure 2e shows the Fourier transform infrared spectroscopy (FTIR) spectra for the pure ZnOX, PbBr_2_+SnBr_2_, and PbBr_2_+SnBr_2_+ZnOX that dissolved in DMSO. It is noted that the stretching vibration of C═O bond shifted from 1661 cm^−1^ in pure ZnOX to a lower wavenumber of 1648 cm^−1^ for the PbBr_2_+SnBr_2_+ZnOX sample. This shift suggests the interaction between oxalate and Pb^2+^ or Sn^2+^ ions, which has been reported to serve as a molecular lock to increase the activation energy of nucleation, thus retarding perovskite crystal growth and improving film crystallization.^[^ ^37^ ^]^ Figure S3 (Supporting Information) compares experimental ^13^C nuclear magnetic resonance (NMR) spectra of pure ZnOX and PbBr_2_+ZnOX in deuterated DMSO‐d6. It is noted that pure ZnOX gives a resonance signal with a chemical shift at 161.83 ppm, which is attributed to oxalate group. However, in PbBr_2_+ZnOX mixture, the corresponding signal downshifted to 161.25 ppm, further verifying the interaction between oxalate and Pb^2+^ or Sn^2+^ ions. The UV–vis absorption results (Figure 2f) of perovskite films reveal a same light absorption edge at around 710 nm. Notably, due to the enhanced crystallinity, the ZnOX film exhibits a stronger absorption along the visible region.^[^ ^38^ ^]^ Further, Urbach tail defects of the films were estimated, and the smaller *E*_u_ of 96.1 meV obtained from ZnOX film shown in Figure S4 (Supporting Information) demonstrates a better crystallized film, which is in well agreement with XRD results. ^[^ ^17^, ^39^, ^40^ ^]^ Figure 2g shows the steady‐state photoluminescence (PL) spectra of the films deposited on quartz glass. The characteristic emission peak of the films located at 712 nm agrees well with the UV–vis results. Besides, the much stronger PL intensity suggest that the nonradiative recombination is significantly suppressed within ZnOX film. Time resolved PL (TRPL) spectra of the films was measured to study charge carrier lifetime, and the results were fitted by a biexponential decay function with detailed parameters summarized in Table S2 (Supporting Information). As presented in Figure 2h, the calculated average carrier life time of the control and ZnOX films was 13.3 and 22.8 ns, respectively. The prolonged carrier life time of the ZnOX film is mainly attributed to the improved crystallinity and suppressed nonradiative recombination.

**Figure 3:**
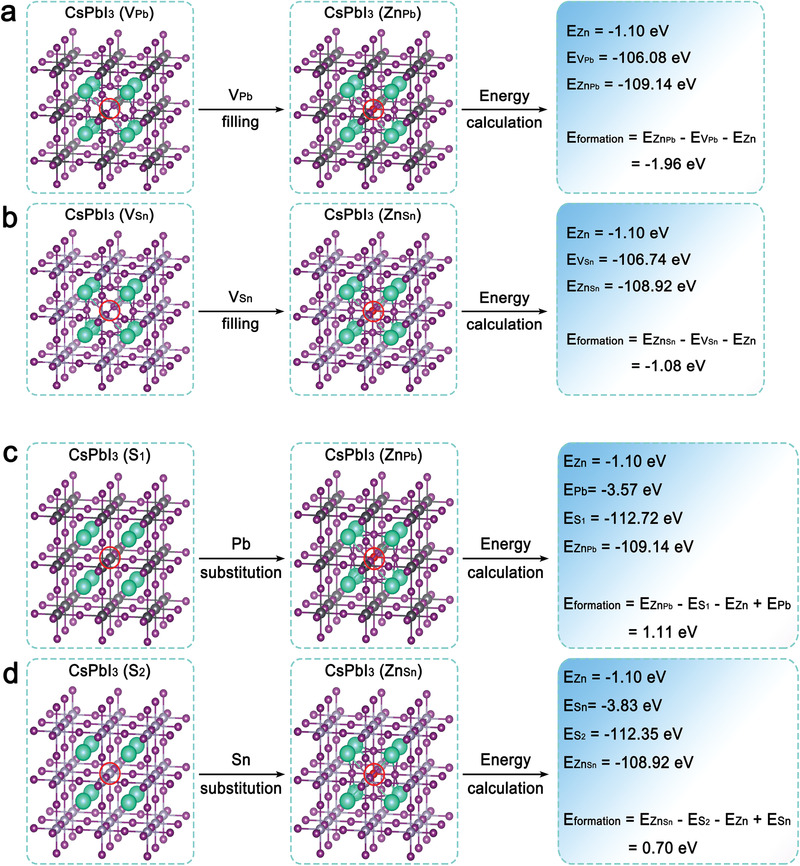
Models and formation energy calculation of a,b) V_Pb_ and V_Sn_ filling and c,d) Pb and Sn substitution.

To have a fully understanding of Zn ions incorporation, two possible incorporation models, V_Pb_ or V_Sn_ filling and Pb or Sn atom substitution, were established via density functional theory (DFT), as presented in **Figure** **3**. 2 × 2 × 2 supercells were adopted for CsPbI_3_ (CsSnI_3_) with Pb (Sn) vacancies and Pb (Sn) vacancies filled with Zn. The cutoff energy of structural relaxation and static calculation was 550 eV. All structures were relaxed until the force on per atom was converged to 0.001 eV Å^−1^. The results reveal that the total formation energies of V_Pb_ and V_Sn_ filling are −1.96 and −1.08 eV, respectively (Figure 3a,b), indicating an exothermic adsorption process. In contrast, Pb and Sn atom substitutions reveal an endothermic adsorption with formation energy of 1.11 and 0.7 eV, respectively (Figure 3c,d). Accordingly, it can be concluded that Zn ions prefer to fill in Pb/Sn vacancies when incorporated into perovskite lattice sites, thus reducing the defect density of perovskite films.

**Figure 4:**
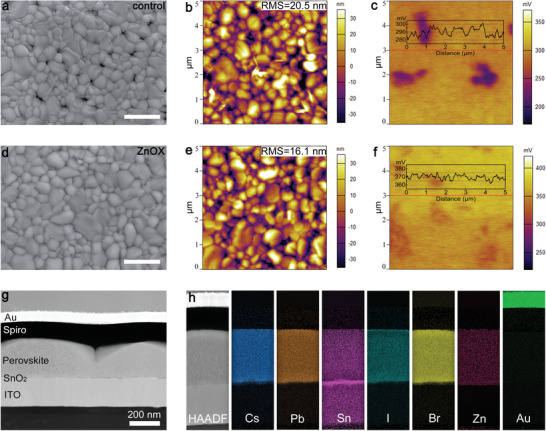
a,d) SEM images, b,e) AFM images, and c,f) KPFM images of control and ZnOX films. The scale bar is 1 µm. g) Cross‐sectional STEM image and h) corresponding EDXS mapping of key elements in HAADF mode for ZnOX film‐based device.

Subsequently, the morphologies and compositional characterizations of the films were also studied to elucidate the effect of ZnOX additive on crystal growth. **Figure** **4**a,d shows the surface SEM images of the perovskites. It is noted that the control film reveals some pinholes and voids in the vicinity of grains (Figure 4a), whereas the ZnOX film exhibits a full coverage of substrate with much larger grain size (Figure 4d). The distinct morphology is mainly attributed to the incorporation of Zn^2+^ ions,^[^ ^35^ ^]^ and the retarded crystallization process due to the strong interaction between oxalate and Pb^2+^ or Sn^2+^ ions, which is further validated by the color change of the as‐deposited perovskites as a function of time (Figure S5, Supporting Information). However, excess ZnOX (over 4%) addition leads to poor morphology and crystal structure distortion with ZnOX segregation at the surface as shown in Figure S6 (Supporting Information). AFM images of the perovskites shown in Figure 4b,e suggest that ZnOX film has a much smoother surface morphology with a root mean square (RMS) of 16.1 nm, which is beneficial for high device performance.^[^ ^41^, ^42^ ^]^ The local surface potential of respective films has been studied using Kelvin probe force microscopy (KPFM) in dark. As shown in the inset of Figure 4c,f, the line profile of ZnOX film reveals a smaller contact potential difference (CPD) variation of 10 mV than that control film (20 mV) across the sample, inferring a mitigation of potential difference between grains and grain boundaries, which can be ascribed to the reduction of defects at the surface due to oxalate coordination.^[^ ^43^, ^44^ ^]^ Moreover, ZnOX introduction leads to an increase of mean CPD in the perovskite film from 290 to 370 mV. It is known that the work function of sample is obtained from the tip work function subtracting the measured CPD value. In this case, the larger CPD value delivers to a smaller work function in ZnOX film, which benefits to charge carrier extraction.^[^ ^45^ ^]^ To examine buried morphology and compositional information, scanning transmission electronic microscopy (STEM) and energy‐dispersive X‐ray spectroscopy (EDXS) mapping was performed. Figure 4g presents the cross‐sectional STEM image of ZnOX film‐based device, in which a uniform stack of functional layers can be observed. The perovskite layer is ≈300 nm thick, and grown continuously on the electron transport layer (ETL) without voids. Corresponding high‐angle annular dark‐field (HAADF) EDXS mapping results are shown in Figure 4h. It can be observed that all compositional elements, such as Cs, Pb, Sn, I, and Br, are distributed homogeneously throughout the entire perovskite film. While, the appearance of Zn element proves the successful alloying of Zn ions in the film. Ultimately, according to the atomic fraction derived from Figure S7 (Supporting Information), the composition of the final perovskite film can be approximately denoted as CsPb_0.7_Sn_0.3_IBr_2_ without regard of minute Zn content, which agrees well with the precursor solution.

**Figure 5:**
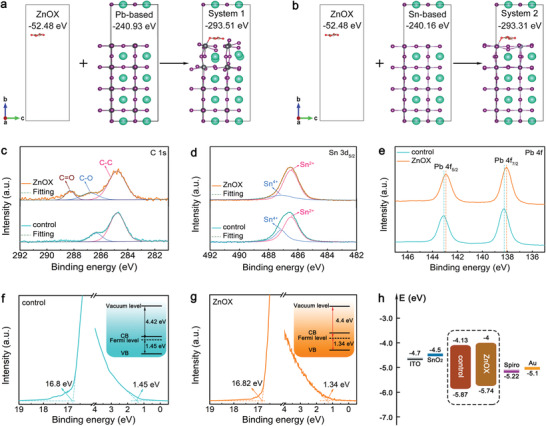
DFT calculation for adsorption energy of oxalate group on a) Pb and b) Sn‐based perovskite. c) C 1s, d) Sn 3d_5/2_, and e) Pb 4f XPS core spectra of control and ZnOX film. UPS spectra of secondary electron cutoff (*E*_cutoff_) and onset (*E*_onset_) energy of the f) control and g) ZnOX film. h) Energy level diagram constructed from UPS results.

To further study the interaction between oxalate group and Pb^2+^ or Sn^2+^ ions, adsorption energy was estimated using density functional theory (DFT) under the level of generalized gradient approximation (GGA) with Perdew–Burke–Ernzerhof (PBE) functional, which was implemented in the Vienna ab‐initio Simulation Package (VASP version 5.4.4.). The adsorption energy (*E*_ad_) was calculated through the following expression

(3) $$E_{\mathrm{ad}}=\;-\left[E_{\mathrm{system}}-\left(E_{\mathrm{adsorbent}}+E_{\mathrm{adsorbate}}\right)\right]$$

where *E*_system_ is the total energy of the optimized system, *E*_adsorbent_ is the total energy of different perovskite, and *E*_adsorbate_ is the total energy of oxalate group. To mimic the adsorption state, a 12.61 × 12.61 Å2 and a 12.41 × 12.41 Å2(001) surface model were built for Pb and Sn‐based perovskite, respectively. A 12‐Å vacuum layer were set to avoid interlayer interaction. A Г‐centered 5 × 5 × 1 k‐point mesh was used to describe the reciprocal space. All the structures were optimized until the force on each atom is smaller than 0.01 eV Å^−1^. Based on the energy values presented in **Figure** **5**a,b, the *E*_ad_ of oxalate group on Sn‐based perovskite is calculated to be 0.67 eV, which is much larger than its Pb‐based counterpart (*E*_ad_ = 0.11 eV). It was reported that a larger *E*_ad_ value means a more stable configuration and exothermic adsorption.^[^ ^46^ ^]^ In this case, the relatively larger *E*_ad_ value suggests that oxalate group shows stronger interaction with Sn‐based perovskite, thus slowing down perovskite crystal growth. With this balanced crystallization rate between Pb and Sn‐based perovskite, highly crystallized mixed Pb‐Sn based CsPb_0.7_Sn_0.3_IBr_2_ perovskite with excellent morphology can be obtained, as shown in Figure 4d.

Chemical compositions and environments of the films were studied by X‐ray photoelectron spectroscopy (XPS), and the corresponding element core spectra are shown in Figure 5c–e and Figure S8 (Supporting Information). As expected, the C 1s core spectrum of ZnOX film reveals clear binding energy peak at 288.3 eV (Figure 5c), which can be indexed to C═O bond of the oxalate group. Besides, the appearance of Zn 2p_3/2_ characteristic peak in ZnOX film (Figure S8a, Supporting Information) indicates the existence of ZnOX in the final perovskite film. The Sn 3d_5/2_ core spectra of the films were deconvoluted into two peaks at 486.5 and 487.2 eV, which are attributed to Sn^2+^ and Sn^4+^, respectively, as presented in Figure 5d.^[^ ^47^, ^48^ ^]^ Clearly, the ZnOX film reveals a much weaker Sn^4+^ peak than that control film, indicating that the introduction of ZnOX can effectively suppress oxidation of Sn^2+^ in mixed Pb–Sn perovskite films, in line with Figure 2b related discussions. In addition, the binding energy shift of the Pb 4f shown in Figure 5e further indicates the interaction between ZnOX and the perovskite film, which is consistent with the FTIR and NMR results (Figure 2e; Figure S3, Supporting Information).

The electronic structures of the films were investigated using ultraviolet photoelectron spectroscopy (UPS). Figure 5f,g shows the obtained secondary electron cutoff (*E*_cutoff_) and onset (*E*_onset_) energy of the control and ZnOX films, respectively. Combining with the optical bandgap derived from UV–vis absorption (inset of Figure 1c), the positions of conduction band minimum (CBM) and valence band maximum (VBM) of the films were ascertained, and the corresponding parameters are summarized in Table S3 (Supporting Information). As depicted in the inset of Figure 5f, the control film is n‐type self‐doped, which might be attributed to the halide vacancies that act as electron donors.^[^ ^49^ ^]^ Interestingly, after introduction of ZnOX, a dedoping process occurred, leading to more intrinsic perovskite film (inset of Figure 5g) which has led to less charge recombination and thus higher device efficiency.^[^ ^50^ ^]^ Additionally, the energy level diagram of the films was constructed based on the UPS results (Figure 5h). It is found that ZnOX film shows an upshift of VBM edge, resulting in a smaller energy gap of 0.52 eV at perovskite/HTL interface than the control film (0.65 eV), which is helpful for collecting the photoexcited holes in the perovskite film with a smaller energy loss.^[^ ^51^ ^]^ It is also worth noting that the energy difference of ZnOX film (0.02 eV) between perovskite/ETL (0.5 eV) and perovskite/HTL (0.52 eV) is much smaller than the control film (0.28 eV). This smaller energy difference contributes to a well‐balanced charge extraction, which is essential for enhancing device performance and mitigating notorious hysteresis.^[^ ^52^ ^]^

**Figure 6:**
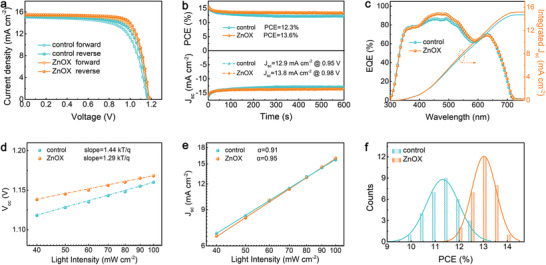
a) The current density–voltage (*J*–*V*) curves, corresponding b) stabilized power outputs and c) EQE of the devices based on control and ZnOX film. Light intensity dependence of d) *V*_oc_ and e) *J*_sc_ for devices based on different perovskite films. f) Statistical PCE parameters of 30 independent PSCs.

To determine the optimal concentration of ZnOX for efficient devices, 0–5% mole ratio of ZnOX was added to the perovskite precursor. Combining with the *J–V* curves (Figure S9, Supporting Information) and parameters summarized in Table S4 (Supporting Information), it is concluded that 2% mole ratio of ZnOX additive delivers optimal device performance, while excess ZnOX additive distorts perovskite crystal structure, thus deteriorating device performance as shown in Figure S6 (Supporting Information). **Figure** **6**a shows the *J–V* curves of the champion PSCs based on control and ZnOX film, and the corresponding photovoltaic parameters are summarized in **Table** **1**. The ZnOX device has an overall enhancement on *V*_oc_, *J*_sc_, and FF, contributing to a PCE as high as 14.1% (reverse scan) which is much higher than 12.9% for the control device. Hysteresis studies based on H‐index (HI): HI  = (PCE_reverse_ − PCE_forward_)/PCE_reverse_ , where PCE_reverse_ and PCE_forward_ are power conversion efficiency of devices for reverse and forward scan, respectively, reveal that ZnOX device delivers a smaller HI (4.2%) than that of control (6.2%), which is mainly attributed to the well‐balanced charge extraction as previously discussed. Note that such a PCE of 14.1% presents the highest efficiency recorded for Br‐rich as well as mixed Pb–Sn based all‐inorganic PSCs thus far, as summarized in **Table** **2**.

**Table 1:** Photovoltaic parameters of champion PSCs based on control and ZnOX film

Device	Scan direction	*V*_oc_ [V]	*J*_sc_ [mA cm^−2^]	FF [%]	PCE [%]	HI [%]
control	Forward	1.16	15.1	69.1	12.1	6.2
	Reverse	1.165	15.1	73.3	12.9	
ZnOX	Forward	1.18	15.4	74.3	13.5	4.2
	Reverse	1.18	15.5	76.7	14.1	

**Table 2:** Comparison with other Br‐rich as well as mixed Pb‐Sn based all‐inorganic PSCs

Compositions	*V*_oc_ [V]	*J*_sc_ [mA cm^−2^]	FF [%]	PCE [%]	Ref.
CsPbIBr_2_	1.29	11.0	78.6	11.2	^[^ ^55^ ^]^
CsPb_0.9_Sn_0.1_IBr_2_	1.26	14.3	63.0	11.33	^[^ ^28^ ^]^
CsPb_0.75_Sn_0.25_IBr_2_	1.21	12.57	75.8	11.53	^[^ ^22^ ^]^
CsPb_0.7_Sn_0.3_IBr_2_	1.18	15.5	76.7	14.1	This work
CsPb_0.7_Sn_0.3_I_3_	0.64	20.96	70.1	9.4	^[^ ^56^ ^]^
CsPb_0.6_Sn_0.4_I_3_	0.774	25.87	66.7	13.37	^[^ ^57^ ^]^
CsSnI_3_	0.64	21.81	72.1	10.1	^[^ ^58^ ^]^

Moreover, to demonstrate the coexistence of effects of Zn ions incorporation and oxalate group interaction with ZnOX additive, ZnI_2_ was adopted to solely study the effect of Zn ions incorporation on crystal structure and device performance as shown in Figure S10 (Supporting Information). Corresponding photovoltaic parameters were summarized in Table S5 (Supporting Information). It is noted that the champion device efficiency derived from ZnI_2_ is 13.4%, which is smaller than that of ZnOX device (14.1%). Accordingly, the efficiency enhancement from 12.9% (control) to 13.4% (ZnI_2_) then to 14.1% (ZnOX) can be attributed to Zn ions incorporation and oxalate group interaction, respectively, demonstrating the multifunction of ZnOX additive.

To confirm the reliability of the *J–V* measurements, steady‐state power output (SPO) at the maximum power point were recorded as shown in Figure 6b. The PCE of the devices based on control and ZnOX film stabilized at 12.3% and 13.6%, respectively, which are close to the values that obtained from the *J–V* curves. External quantum efficiency (EQE) spectra of the devices are presented in Figure 6c, where ZnOX film‐based device shows stronger light response during the visible region, in accordance with the UV–vis absorption results (Figure 2f). The integrated *J*_sc_ located at 14.7 and 15.2 mA cm^−2^ for the devices based on control and ZnOX film, respectively, which are in good agreement with the measured value. In addition, dependence of *V*_oc_ and *J*_sc_ on light intensity measurements were conducted to study charge extraction and recombination in the devices. As shown in Figure 6d, ZnOX film‐based device exhibits a smaller slope of 1.29 kT q^−1^ than that of control device (1.44 kT q^−1^). It has been reported that the deviation of the slope from unity kT q^−1^ indicates the trap‐assisted recombination in PSCs.^[^ ^6^, ^53^ ^]^ Accordingly, the smaller slope suggests that the trap‐assisted recombination within ZnOX film was substantially reduced, which is responsible for the enhancement of *V*_oc_. Besides, it is noted that both the control and ZnOX film‐based devices show a linear *J*_sc_ versus light intensity relationship (Figure 6e), indicating a favorable environment for charge extraction.^[^ ^11^, ^54^ ^]^ While a more ideal *α* value (0.95) for ZnOX film suggests the formation of high‐quality perovskite film with better energy level alignment between perovskite and charge transport layer, thus facilitating charge extraction and collection. Apart from enhanced device performance, the reproducibility of the ZnOX film‐based PSCs was also improved. Figure 6f presents the statistical histogram of the PCEs derived from 30 independent PSCs. Being different from the wide PCE distribution (10% to 12.5%) of control devices, ZnOX film‐based devices produce a narrower PCE distribution ranging from 12% to 14%. This highly improved reproducibility is mainly ascribed to the better crystallized ZnOX film with suppressed Sn^2+^ oxidation.

**Figure 7:**
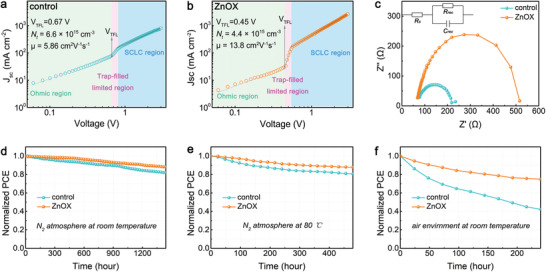
Dark *J*–*V* curves for the electron‐only devices based on a) control and b) ZnOX film. c) Nyquist plots and equivalent circuit for the different devices. Stability measurements of the unencapsulated PSCs based on control and ZnOX films in d) glovebox at room temperature, e) glovebox at 80 °C, and f) air environment at room temperature.

To better understand how ZnOX improve the device efficiency, space‐charge‐limited current (SCLC) technique was adopted to evaluate the defect density of perovskite films. **Figure** **7**a,b illustrates the typical dark *J–V* curves of electron‐only devices with structure of ITO/SnO_2_/Perovskite/PCBM/Ag. Notably, ZnOX film‐based device shows a smaller trap‐filled limit voltage (*V*_TFL_) of 0.45 V than its control film‐based counterpart (0.67 V). It is well known that the *V*_TFL_ is closely related to defect density, which can be expressed by

(4) $$N_{t}=\frac{2V_{\mathrm{TFL}}\varepsilon_{r}\varepsilon_{0}}{eL^{2}}\;$$

where *ε*_r_ is the relative dielectric constant of perovskite, which is ≈8,^[^ ^59^ ^]^ *ε*_0_ is the vacuum permittivity, *e* is the elementary charge, and *L* is the thickness of the perovskite film, which is ≈300 nm according to Figure 4g. Accordingly, the calculated defect density of control and ZnOX films are 6.6 × 10^15^ and 4.4 × 10^15^ cm^−3^, respectively. The substantially reduced defect density of ZnOX film is associated with the reduction of V_Pb_, V_Sn_ and undercoordinated metal ions, as discussed earlier. Further, the electron mobility of the films was estimated using the Mott–Gurney equation as follows

(5) $$\mu \;=\frac{8J_{D}L^{3}}{9\varepsilon_{r}\varepsilon_{0}V^{2}}\;$$

where *J*_D_ and *V* are current density and voltage at the SCLC region, respectively.^[^ ^60^, ^61^ ^]^ By introducing ZnOX, the electron mobility was increased from 5.86 to 13.8 cm^2^ V^−1^ S^−1^, which is ascribed to the improved crystallinity. Furthermore, the interfacial charge transfer and recombination were investigated using electrochemical impedance spectroscopy (EIS) measurement. Nyquist plots of the devices based on different films were measured under a bias of 1 V in the frequency range of 1 MHZ to 10 HZ under dark condition at room temperature (Figure 7c). The larger semicircle derived from ZnOX film‐based device correspond to a larger recombination resistance (*R*_rec_), inferring that the charge recombination process is effectively suppressed within the device.^[^ ^62^ ^]^ Overall, the less defect density, higher electron mobility coupled with suppressed charge recombination are seen as the main reasons for the enhanced photovoltaic performance in the ZnOX film‐based device.

Besides the device efficiency, stability is another critical concern for PSCs. To demonstrate the positive effect of ZnOX on device stability, long‐term, thermal, and air stability for the unencapsulated devices were investigated, and the corresponding PCE decay was presented in Figure 7d–f. Clearly, ZnOX film‐based device exhibits superior stability over control device under the same condition. Particularly, a remarkable enhancement on air stability can be observed from Figure 7f, where ZnOX film‐based device retains over 75% of its original PCE after 10 d exposure to air, whereas the control device quickly drops to 42% of its initial value. This significant improvement of stability can be attributed to three points. First, the excellent film morphology with large grains and less grain boundaries reduce the pathways for moisture and oxygen ingress, thus suppressing film degradation.^[^ ^63^ ^]^ Second, the strong interaction between oxalate group and perovskite can inhibit ion migration during heating, thereby enhancing the thermal stability. Third, chemically reducing oxalate group can effectively suppress the oxidation of Sn^2+^ ions, which is essential for the air stability of the devices.

## 3 Conclusion

In conclusion, we have introduced ZnOX as multifunctional additive to fabricate highly efficient and stable mixed Pb–Sn based CsPb_0.7_Sn_0.3_IBr_2_ all‐inorganic perovskite solar cells. It was found that this novel additive not only minimize the formation of V_Pb_ and V_Sn_ through Zn^2+^ ions incorporation but also modulate film crystallization via interaction with undercoordinated metal ions. Consequently, perovskite films with low defect density, high crystallinity, and superior charge dynamic properties are obtained. As a result, a PCE as high as 14.1%, which presents the highest reported efficiency for bromine‐rich as well as mixed Pb–Sn based all‐inorganic PSCs thus far, was achieved. Besides, chemically reducing oxalate group can effectively mitigate the oxidation of Sn^2+^ ions, resulting in devices with significantly improved long‐term air stability. With the mentioned outstanding advantages, it is believed that appropriate oxalate compound additive can be extended toward air‐stable mixed Pb–Sn or lead‐free Sn‐based PSCs.

## Conflict of Interest

The authors declare no conflict of interest.

## Supporting information

Supporting Information

## Data Availability

The data that support the findings of this study are available from the corresponding author upon reasonable request.
