# Supplementary material for: Oxalate Pushes Efficiency of CsPb0.7Sn0.3IBr2 Based All‐Inorganic Perovskite Solar Cells to over 14%
Source: Adv Sci (Weinh). 2022 Feb 12;9(11):2106054. doi: 10.1002/advs.202106054 (PMC9009130; doi:10.1002/advs.202106054)
Supplement: Supplementary file 1 — Supporting Information [file ADVS-9-2106054-s001.pdf]

WILEY-VCH

ADVANCED SCIENCE
Open Access

Supporting Information

for *Adv. Sci.*, DOI: 10.1002/advs.202106054

Oxalate Pushes Efficiency of $CsPb_{0.7}Sn_{0.3}IBr_2$ Based All-Inorganic Perovskite Solar Cells to over 14%

*Weihai Zhang, Heng Liu, Xingnan Qi, Yinye Yu, Yecheng Zhou, Yu Xia, Jieshun Cui, Yueqing Shi, Rui Chen, and Hsing-Lin Wang**

Supporting Information

Oxalate Pushes Efficiency of $CsPb_{0.7}Sn_{0.3}IBr_2$ Based All-Inorganic Perovskite Solar Cells to over 14%

*Weihai Zhang, Heng Liu, Xingnan Qi, Yinye Yu, Yecheng Zhou, Yu Xia, Jieshun Cui, Yueqing Shi, Rui Chen, and Hsing-Lin Wang**

Dr. Weihai Zhang, Heng Liu, Xingnan Qi, Yu Xia, Dr. Jieshun Cui, Prof. Hsing-Lin Wang
Department of Materials Science and Engineering,
South University of Science and Technology,
Shenzhen, 518055, China
E-mail: wangxl3@sustech.edu.cn

Yinye Yu, Prof. Yecheng Zhou
School of Materials Science and Engineering,
Sun Yat-sen University,
Guangzhou, 510275, China

Yu Xia
School of Physics and Astronomy,
University of Birmingham,
Edgbaston, Birmingham, UK

Yueqing Shi, Prof. Rui Chen
Department of Electrical and Electronic Engineering,
South University of Science and Technology,
Shenzhen, 518055, China

Experimental Section

Materials: The ITO substrates and quartz glasses were purchased from Advanced Election Technology Co., Ltd. The SnO_2 colloid, Znic Oxalate (ZnOX), and Tin Fluoride (SnF_2, 99.9%) were purchased from Alfa Aesar. Cesium iodide (CsI), Lithium-bis (trifluoromethanesulfonyl) imide (Li-TFSI), FK209 Co(III) TFSI salt, and Spiro-OMeTAD were purchased from Xi'an Polymer Light Technology Corp. Lead bromide ($PbBr_2$, 99.999%), Tin bromide ($SnBr_2$, 99.9%), Dimethyl sulfoxide (DMSO, 99.9%), and Isopropanol (99.5%) were purchased from Sigma-Aldrich.

Device Fabrication: ITO glass substrates (7 Ω sq^{-1}) were thoroughly cleaned and treated with plasma for 5 min before usage. Then, a layer of SnO_2 was spin-coated at 3500 rpm for 30 s and annealed at 150 °C for 30 min in ambient air. After cooling to room temperature, the substrates were transferred to a nitrogen filled glove box. All of the perovskite films were spin-coated from a precursor solution containing CsI (1 M), $PbBr_2$ (*1-x* M), $SnBr_2$ (*x* M), and SnF_2 (0.1*x* M) in DMSO, with a spin protocol of 1000 rpm for 3 s and then at 4000 rpm for 30 s. Different content of ZnOX from a stock solution (1 M in HBr:DMSO=1:4) was directly added into perovskite precursor during precursor preparation. With SAG method, the corresponding as-deposited perovskite films were treated by spin-coating 150 μL of MAI solution (10 mg/mL in isopropanol) at 4000 rpm for 15 s. After that, the films were annealed at 60 °C for 1 min and then at 160 °C for 10 min. After cooling to room temperature, a conventional Spiro-OMeTAD layer, which consisted of 72.3 mg spiro-OMeTAD, 28.8 μL 4-tertbutylpyridine, 17.5 μL lithium-bis (trifluoromethanesulfonyl) imide (Li-TFSI) solution (520 mg Li-TFSI in 1 mL acetonitrile), 10 μL FK209 Co(III) TFSI solution (300 mg in 1 mL acetonitrile), and 1 mL chlorobenzene, was deposited on the perovskite films as the hole transport layer. Finally, 80 nm Au was fabricated as the electrode using a shadow mask under high vacuum by thermal evaporation. The active area of the devices was 0.06 cm^2.

Characterizations: The XRD patterns (2θ scans) were obtained on Bruker Advanced D8 X-ray diffractometer using Cu Kα (λ = 0.154 nm) radiation. A UV-Vis spectrophotometer (Agilent Cary 5000) was used to collect the absorbance spectra of the perovskite films. Steady state photoluminescence (PL) spectra were recorded on Shimadzu RF-5301pc. Time-resolved photoluminescence spectra were measured on a PL system (Fluo-Time 300) under excitation with a picosecond pulsed diode laser at 520 nm wavelength with a repetition frequency of 1 MHz. The ^{13}C nuclear magnetic resonance (NMR) spectra were recorded in deuterated dimethyl sulfoxide (DMSO-d6) using a 300 MHz Bruker spectrometer. The morphology of the films was studied by field-emission scanning electron microscopy (TESCAN, MIRA3)

and atomic force microscopy (AFM, Asylum Research MFP-3D-Stand Alone). An FEI Helios Nanolab 600i dual beam, focus ion beam/field emission gun-scanning electron microscope (FIB/FEGSEM) (FEI, Netherland), was used to prepare the device cross-section for scanning transmission electronic microscopy (STEM) imaging and analysis (FEI, Netherland). FEI Talos transmission electron microscope (TEM) with Super-X energy dispersive X-ray EDX was employed to acquire the STEM-EDX data with STEM high-angle annular dark field (STEM-HAADF) mode. X-ray photoelectron spectroscopy (XPS) was conducted on a Thermo Scientific™ K-Alpha™+ spectrometer equipped with a monochromatic Al Kα X-ray source (1486.6 eV) operating at 100 W. Samples were analyzed under vacuum ($P < 10^{-8}$ mbar) with a pass energy of 150 eV (survey scans) or 50eV (high-resolution scans). All peaks were calibrated with C 1s peak binding energy at 284.8 eV for adventitious carbon. Ultraviolet photoelectron spectroscopy (UPS, ESCALAB 250Xi, Thermo Fisher) measurements were carried out using a He I discharge lamp (21.22 eV). Current density-voltage (J-V) curves of the devices were collected using a source meter (Keysight B2901A) and a solar simulator (Enlitech SS-F5-3A) with a protocol of 1.4 to -0.1 V with a 20 mV voltage step and 200 ms delay. The light intensity was calibrated to AM 1.5G (100 mW cm^{-2}) by using a reference Si solar cell. The external quantum efficiency (EQE) spectra were recorded with a quantum efficiency measurement system (Enlitech QER-3011) in which the light intensity at every wavelength was calibrated with a Si detector before measurement. The maximum-power point (MPP) output was measured by testing the steady-state current density at the maximum-power-point voltage. Electrochemical impedance spectroscopy (EIS) was performed in the frequency range from 10 Hz to 1 MHz by an electrochemical workstation (Princeton Applied Research, P4000+) in dark conditions with a bias of 1 V. The amplitude is 10 mV.

Statistical Analysis: In order to facilitate comparison, part of the data is normalized, such as the time-resolved PL (TRPL) results in Figure 2h and the stability results in Figure 7d-f. The statistical results were obtained by presenting the photovoltaic parameters of different number of devices. The sample size, specific test, and data presentation of each statistical analysis was specified in the corresponding figure legend. The TRPLcurves were fitted using OriginLab software. The scale bars of SEM, TEM and AFM images (Figure 4) were presented in the corresponding figure and specified in the figure legend. Specific details for all methods and softwares are discussed in the Characterization Section.

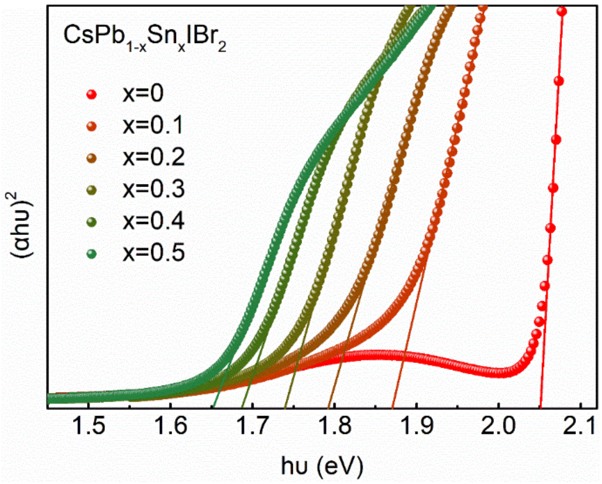

Figure S1. Tauc's plot calculated from the UV-Vis absorption spectra with equation $(\alpha h\nu)^2 = A(h\nu - E_g)$, where α is the absorption coefficient, hυ is the photon energy, and E_g is the bandgap.

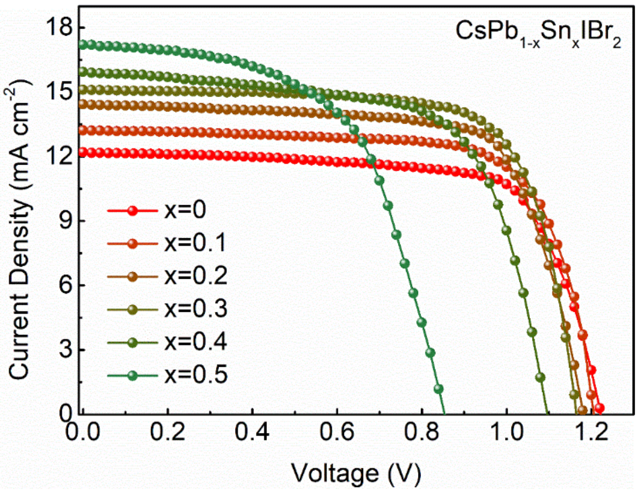

Figure S2. J-V curves of champion PSCs based on film with different Sn concentration (*x* value). It is noted that with the increase of *x* value, the devices show decrease in V_{oc}, and increase in J_{sc}, which is ascribed to the reduced bandgap.

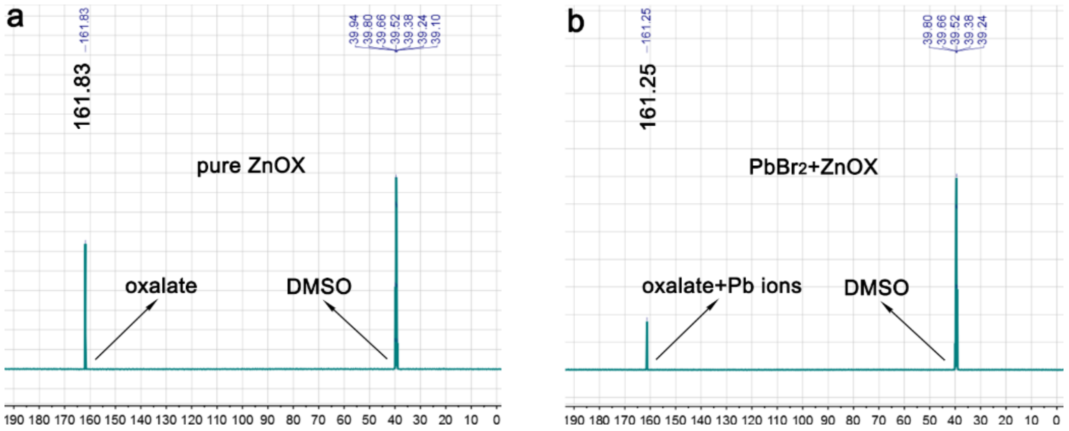

Figure S3. ^{13}C NMR spectra of a) pure ZnOX and b) $PbBr_2$+ZnOX. The downshifted resonance sigaml of oxalate from 161.83 ppm for pure ZnOX to 161.25 ppm for mixed $PbBr_2$+ZnOX is mainly attributed to the interaction between oxalate and Pb^{2+} ions.

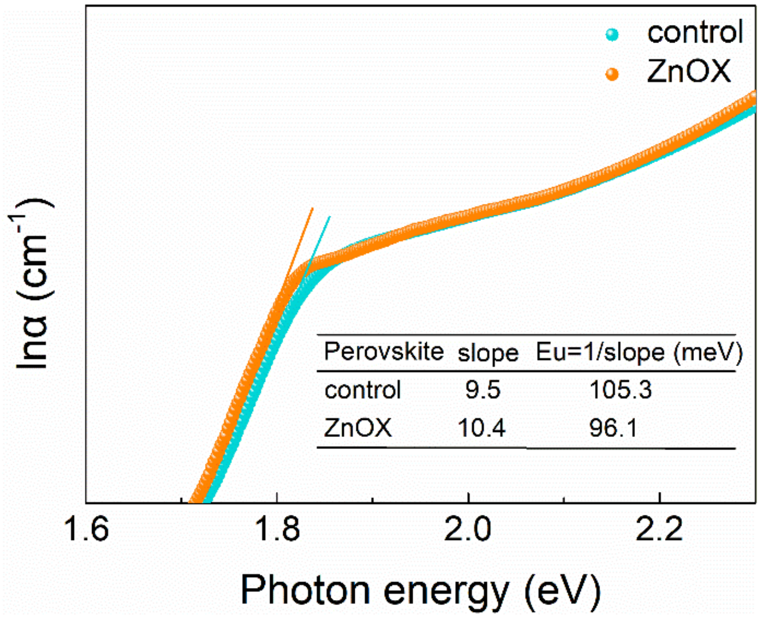

Figure S4. Urbach energy calculated from the UV-Vis absorption spectra with equation $ln\alpha = ln\alpha_0 + (h\upsilon/E_u)$, where α is the absorption coefficient, hυ is the photon energy, and E_u is the Urbach energy. As is reported,[1,2] low Eu is highly desirable for semiconductor devices, indicative of highly crystallized film with less impurities.

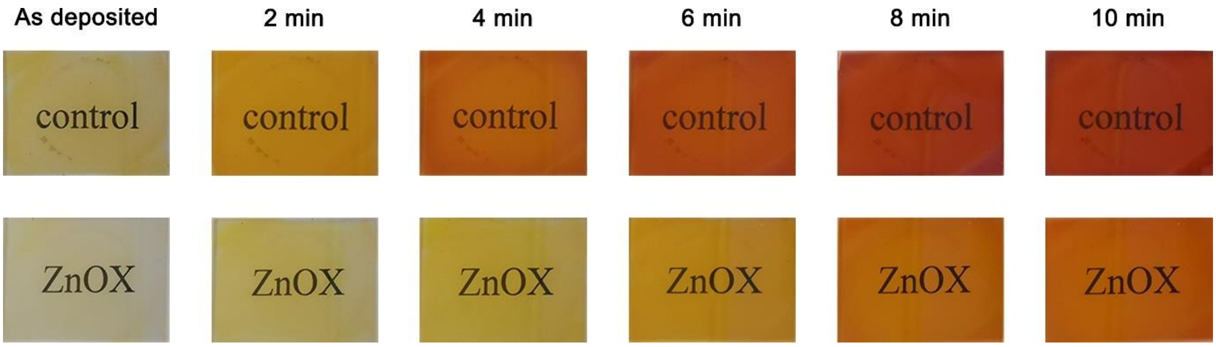

Figure S5. Photographs of as-deposited control and ZnOX films at different stage before annealing treatment. It can be seen that the surface color of control film quickly changes from colorless to redbrown within 4 min, while ZnOX film reveals a significant mitigation of color change from colorless to lightyellow (4 min) then to redbrown (10 min). The distinct color change indicates that ZnOX can effectively retard the nuclei formation of perovskite film.

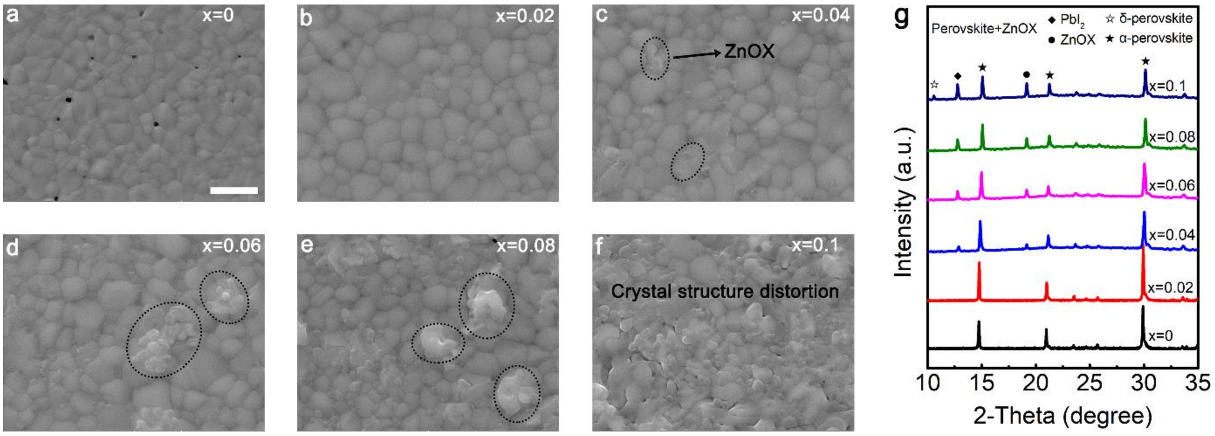

Figure S6. SEM and XRD results of perovskite films with different content of ZnOX. The scale bar is 1 μm. It can be seen that moderate ZnOX addtive (2%) improves the morphology of the perovskite with full coverage of substrate and large grains. However, excess ZnOX additive (over 4%) leads to ZnOX segragation at the surface. When, the content comes to 10%, crystal structure distortion with poor crystallinity can be clearly observed, as supported by XRD results.

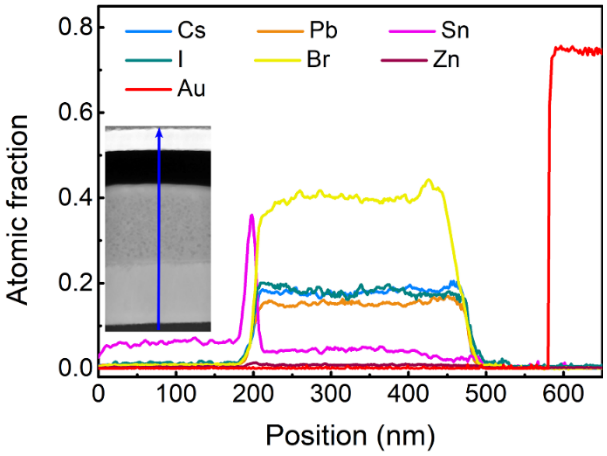

Figure S7. EDXS line profile with Cs, Pb, Sn, I, Br, Zn, and Au atomic fraction extracted from the blue arrow from bottom to top. The region from 200 to 500 nm corresponds to perovskite layer. According to respective atomic fraction (Cs=18%, Pb=13%, Sn=5%, I=18%, Br=38%, and Zn=0.7%), the composition of the final perovskite film can be approximately denoted as $CsPb_{0.7}Sn_{0.3}IBr_2$ without regard of minute Zn content.

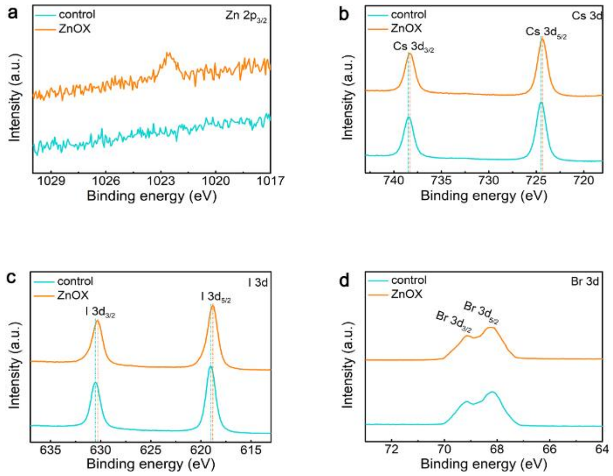

Figure S8. a) Zn $2p_{3/2}$, b) Cs 3d, c) I 3d, and d) Br 3d XPS core spectra of control and ZnOX film.

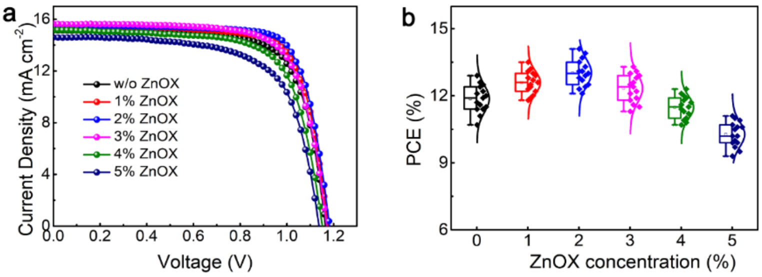

Figure S9. a) J-V curves of PSCs based on film with different ZnOX concentration. b) Statistical PCE results of 15 independent PSCs based on film with different ZnOX concentration.

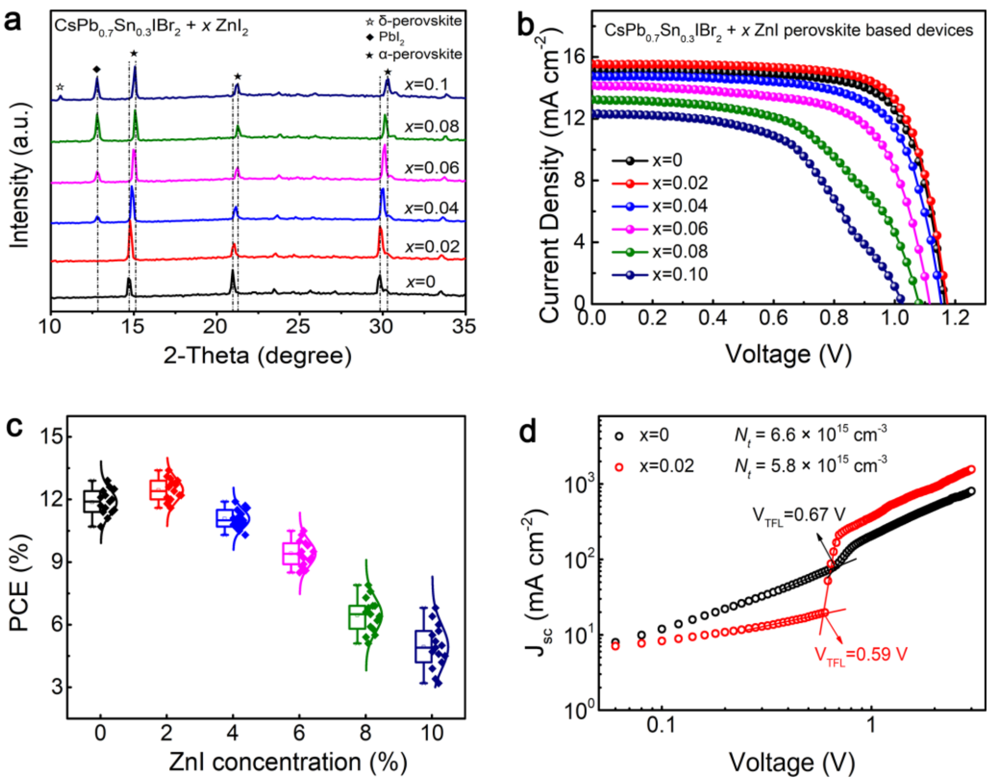

Figure S10. a) XRD patterns of $CsPb_{0.7}Sn_{0.3}IBr_2$ perovskite film with different content of ZnI_2 addition, b) J-V curves of PSCs based on film with different content of ZnI_2 addition, c) statistical PCE results of 15 independent PSCs based on film with different ZnI_2 concentration, d) dark J–V curves for the electron-only devices based perovskite film with 2% and without ZnI_2 addition.

The XRD results shown in Figure S10a reveal a peak shift toward higher angles with the increase of ZnI_2 concentration, suggesting the incorporation of Zn ions. When the ZnI_2 content increased to over 2%, the crystallinity of the perovskite is degraded with the appearance of PbI_2 and δ-phase perovskite diffraction peaks, which is attributed to the structure distortion induced by the smaller Zn ions. The J-V curves and statistical photovoltaic parameters suggest that moderate ZnI_2 addition (2%) delivers an enhancement of device efficiency, while excess ZnI_2 additive deteriorates device performance as summarized in Table S5. Further, space-charge-limited current (SCLC) technique was adopted to evaluate the defect density of the perovskite films, demonstrating a reduction of defect density from 6.6×10^{15} cm^{-3} to 5.8×10^{15} cm^{-3} after 2% ZnI_2 addition, which is mainly derived from Pb and Sn vacancies filled by Zn ions.

Table S1. Photovoltaic parameters of champion PSCs based on $CsPb_{1-x}Sn_xIBr_2$ film with different x value.

x value	V_{oc} [V]	J_{sc} [mA cm^{-2}]	FF [%]	PCE [%]
0	1.23	12.1	72.2	10.7
0.1	1.20	13.2	72.7	11.5
0.2	1.18	14.4	72.4	12.3
0.3	1.16	15.1	73.0	12.8
0.4	1.08	15.9	67.5	11.6
0.5	0.85	17.2	57.2	8.4

Table S2. Fitted TRPL parameters of different perovskite films.

Perovskite	A_1 [%]	τ_1 [ns]	A_2 [%]	τ_2 [ns]	τ_{ave} [ns]
control	58.08	17.7	41.92	7.3	13.3
ZnOX	54.92	32.2	45.08	11.6	22.8

Note: The fitting biexponential decay function was $y = y_0 + A_1 exp\left(-\frac{t}{\tau_1}\right) + A_2 exp\left(-\frac{t}{\tau_2}\right)$.

Table S3. Electronic parameters of different perovskite films derived from UPS spectra.

Perovskite	E_{cutoff} [eV]	E_{onset} [eV]	VBM [eV]	E_g [eV]	CBM [eV]
control	16.8	1.45	5.87	1.74	4.13
ZnOX	16.82	1.34	5.74	1.74	4

Table S4. Photovoltaic parameters of PSCs based on film with different ZnOX concentration.

ZnOX content [%]	V_{oc} [V]	J_{sc} [mA cm^{-2}]	FF [%]	PCE [%]
w/o	1.165	15.1	73.3	12.9
1	1.18	15.4	74.3	13.5
2	1.18	15.5	76.7	14.1
3	1.175	15.6	72.5	13.3
4	1.155	15.2	70.4	12.3
5	1.14	14.6	66.7	11.1

Table S5. Photovoltaic parameters of PSCs based on film with different ZnI_2 concentration.

ZnI_2 content (%)	V_{oc} [V]	J_{sc} [mA cm^{-2}]	FF [%]	PCE [%]
w/o	1.165	15.1	73.3	12.9
2	1.175	15.5	73.6	13.4
4	1.155	14.8	69.6	11.9
6	1.12	14.2	66.0	10.5
8	1.085	13.3	54.7	7.9
10	1.035	12.3	53.4	6.8

References

[1] Y. Chen, N. Li, L. Wang, L. Li, Z. Xu, H. Jiao,P. Liu, C. Zhu, H. Zai, M. Sun, W. Zou, S. Zhang, G. Xing, X. Liu, J. Wang, D. Li, B. Huang, Q. Chen, H. Zhou, *Nat. Commun.* **2019**, *10*, 1112.

[2] A. Rajagopal, P. W. Liang, C. C. Chueh, Z. Yang, A. K. Y. Jen, *ACS Energy Lett.* **2017**, *2*, 2531.
